# Prolonged Peripheral Immunosuppressive Responses as Consequences of Random Amphetamine Treatment, Amphetamine Withdrawal and Subsequent Amphetamine Challenges in Rats

**DOI:** 10.1007/s11481-021-09988-1

**Published:** 2021-02-13

**Authors:** Wojciech Glac, Joanna Dunacka, Beata Grembecka, Grzegorz Świątek, Irena Majkutewicz, Danuta Wrona

**Affiliations:** Department of Animal and Human Physiology, Faculty of Biology, University of Gdansk, 59 Wita Stwosza Str, 80-308 Gdansk, Poland

**Keywords:** Chronic random amphetamine treatment, Withdrawal, Amphetamine challenge after withdrawal, Rats, Inflammatory response, Drug abuse

## Abstract

Drug-induced immunosuppression may underline increased hypothalamic–pituitary–adrenal axis response to stress observed following chronic psychostimulant treatment. However, the consequences of random amphetamine (AMPH) treatment, withdrawal and AMPH challenge after withdrawal on the peripheral immunity and systemic corticosterone response are unknown. In this study, the total blood and spleen leukocyte, lymphocyte, T, B, NK, TCD4^+^/TCD8^+^ cell numbers and ratio, pro-inflammatory interferon gamma (IFN-γ), and anti-inflammatory interleukin-4 (IL-4) production, and plasma corticosterone concentration in Wistar rats were investigated after: chronic, random AMPH/SAL treatment alone (20 injections in 60 days, 1 mg/kg b.w., i.p.), AMPH/SAL withdrawal (for 20 consecutive days after random AMPH/SAL exposure) or AMPH/SAL challenge after withdrawal (single injection after the AMPH/SAL withdrawal phase). The results showed blood and spleen leukopenia, lymphopenia, lower blood production of IFN-ɤ, and increased plasma corticosterone concentration after the AMPH treatment, which were more pronounced in the AMPH after withdrawal group. In contrast, an increased number of blood NK cells and production of IL-4 after chronic, random AMPH treatment alone, were found. Blood AMPH-induced leukopenia and lymphopenia were due to decreased total number of T, B lymphocytes and, at least in part, of granulocytes and monocytes. Moreover, decreases in the number of blood TCD4^+^ and TCD8^+^ lymphocytes both in the AMPH chronic alone and withdrawal phases, were found.

The major findings of this study are that AMPH treatment after the long-term withdrawal from previous random AMPH exposure, accelerates the drug-induced immunosuppressive and systemic corticosterone responses, suggesting prolonged immunosuppressive effects and an increase in incidence of infectious diseases.

**Graphical Abstract:**

Prolonged peripheral immunosuppressive responses as consequences of random amphetamine…The results indicate that the chronic and random AMPH exposure alone and the acute (single injection) challenge of the drug after the withdrawal phase induced long-term immunosuppressive effects, which were similar to those occurring during the stress response, and sensitized the peripheral immunosuppressive and corticosterone responses of the rat to the disinhibitory effects of this stressor.

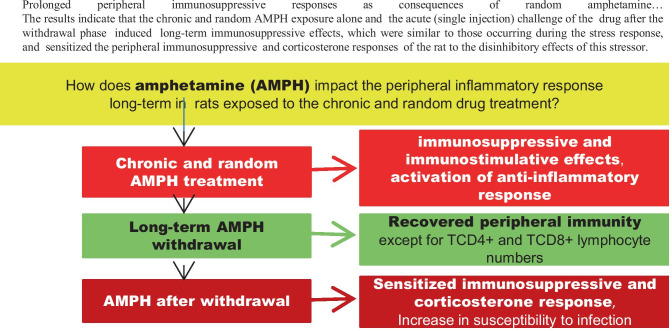

## Introduction

The drug addiction is a chronically relapsing disorder characterized by compulsive drug use and loss of control over drug intake (Kolokotroni et al. 2012; Wemm and Sinha 2019). The psychostimulants amphetamine (AMPH) and methamphetamine (METH) are potent and addictive illicit drugs that are frequently abused worldwide. Chronic AMPH/METH use can result in serious and pronounced cognitive (Sahaklan et al. 2000), neurological (Volkow et al. 2001) and psychiatric dysfunctions (Dyer and Cruickshank 2007; Huckans et al. 2017) in addition to physical health problems (Turnipseed et al. 2003). The reinforcing properties of AMPH/METH are associated with prolonged and enhanced functionality monoamine neurotransmitter dopamine and noradrenaline within the mesocorticolimbic circuit (Goodman et al. 1990; Meredith et al. 2005; Barr et al. 2010). Discontinuation of chronic use of the AMPH/METH may produce a withdrawal syndrome and symptoms resembling endogenous depression (Barr et al. 2010; Shabani et al. 2019).

Comparatively few animal studies have investigated effects of chronic AMPH administration on immune system function. However, there is growing evidence that AMPH suppresses and modulates the immune system (Kohno et al. 2019; Papageorgiou et al. 2019). AMPH has the capacity to modulate immune cells resulting in the drug long-term effects which may manifests as a neuropsychiatric disorder and that increases susceptibility to such diseases as HIV, infections (Freire-Garabal et al. 1999) as well as tumors (Freire-Garabal et al. 1992). AMPH-induced changes in the cytokine balance have been associated with compromise the blood–brain barrier, leading to alterations in brain plasticity and creating lasting neurotoxicity (O’Callaghan and Miller 1994; Bowyer 2014). Recent reports suggest that AMPH has significant effects on both the innate and adaptive immune responses (Bowyer et al. 2015; Camacho et al. 2019), with reported reductions in the numbers of natural killer (NK) cells and leukocytes, immunosuppressive effects on the T cell-mediated immune response, diminished generation of cytotoxic T lymphocytes (Nunez-Iglesias et al. 1996) and proliferative response of splenocytes (Freire-Garabal et al. 1991; Kubera et al. 2002). In contrast, we previously reported (Wrona et al. 2005) that single AMPH injection (1 mg/kg b.w., i.p.) in rats increased blood NK cell number and cytotoxic activity accompanied by lymphopenia, neutrocytosis, monocytosis, and an increased plasma corticosterone concentration. This immunostimulative AMPH-induced effect on anti-tumor activity of NK cells was dependent on beta-adrenergic mechanism (Glac et al. 2006). In addition, macrophages stimulated by AMPH showed increased levels of the pro-inflammatory cytokine TNF-α while the adaptive immune system was decreased and rendering individuals susceptible to certain diseases and infection (Levine et al. 2014). Faster progression of HIV infection and increased incidence of acquired immunodeficiency syndrome (AIDS) in addicts suggests that AMPH use results in a functional impairment of the immune systems, which selectively involves the peripheral blood lymphocyte subsets, in particular, the TCD4^+^/TCD8^+^ lymphocyte ratio. The TCD4^+^/TCD8^+^ lymphocyte ratio in blood is used in the diagnosis of HIV infections (Pahwa et al. 2008), hepatitis C virus (Viso et al. 2007) and autoimmune disorders (Pichler et al. 2009).

In view of these considerations and given the limited research exploring the chronic effects of random AMPH treatment on peripheral inflammatory status, the purpose of the present study was to evaluate consequences of random AMPH administration in rats after three phases of the experiment: 1) chronic, random AMPH treatment alone phase (20 injections of AMPH in 60 days in a moderate dose of 1 mg/kg), 2) late AMPH withdrawal phase (AMPH withdrawal group, withdrawal for 20 consecutive days after AMPH treatment termination) and 3) sensitivity to acute (single injection) AMPH challenge following the prolonged withdrawal (AMPH after withdrawal group). In order to better understand the effects played by AMPH-induced immunomodulation during the drug maintenance and the drug challenge after withdrawal, we have also assessed these effects during the late phase of the drug withdrawal. The peripheral immune parameters examined were as follows: changes in the numbers of blood and spleen leukocytes, T (CD3^+^), B (CD45RA^+^), NK (CD161a^+^), TCD4^+^/TCD8^+^ lymphocyte subsets, TCD4^+^/TCD8^+^ ratio and mitogen-induced release of such pro-/anti-inflammatory cytokines as interferon – γ (IFN-γ) and interleukin 4 (IL-4). Production of IFN-ɤ and IL-4 in blood was measured as a marker of AMPH-related activity of T CD4^+^ and NK cells in peripheral blood, and susceptibility to infection. Moreover, circulating corticosterone as a potent regulator of the immune response and a marker of the stress response and the activity of HPA axis, was determined. As far as we know, effects of AMPH challenge followed by the chronic, random exposure to this drug with a subsequent withdrawal from AMPH on peripheral inflammatory and systemic corticosterone responses in rats, have not been reported before.

## Experimental Procedure

### Animals

**Fig. 1:**
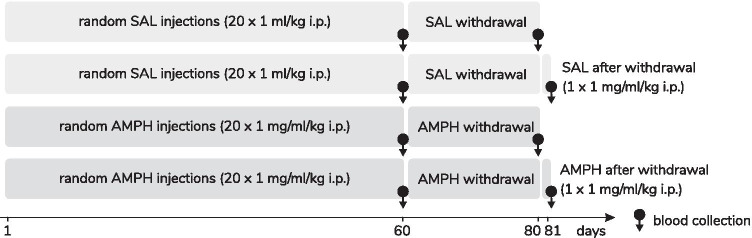
Diagram of experimental procedures and group assignments. Explanations: random AMPH/SAL injections–20 injections (one per day, i.p.) of AMPH or SAL in a dose of 1 mg/ml/kg b.w. during randomly chosen 20 days in 60 days of the chronic, irregular AMPH/SAL treatment alone; AMPH/SAL withdrawal – AMPH or SAL withdrawal for 20 consecutive days after the chronic, irregular AMPH/SAL treatment; AMPH/SAL after withdrawal – single injection of AMPH or SAL (i.p.) in a dose of 1 mg/ml/kg b.w. after AMPH/SAL withdrawal; blood collection – blood was collected twice from all the AMPH treated and SAL groups: one hour after the last injection of AMPH or SAL (on the 60 day of the experiment) and one day after single AMPH or SAL injections (on the 81^st^ day of the experiment, 09.00 a.m.)

Adult male Wistar rats, purchased from a licensed breeder (Three-City Laboratory Animals Breeding, Gdańsk, Poland), weighing 250 ± 20 gat the beginning of the experimental procedure, were used. Animals were caged in groups of 3, with free access to food and water. Animal room was maintained at 22ºC, under 12 h light/12 h dark illumination cycle (on 6 am / off 6 pm). To assess effects of the AMPH treatment and withdrawal phase on the peripheral blood immune parameters and plasma corticosterone concentration, the AMPH- and saline treated rats were housed at the dark and light cycle, with all experiments conducted during the light cycle. For seven days rats were handled and adapted to the presence of the experimenter to minimalize stress evoked by experimental procedures. Figure 1 shows a diagram with the experimental procedure. The animals were divided randomly into four groups: the AMPH withdrawal group (n = 10; withdrawal from the chronic, random drug exposure), that was previously subjected to 20 injections ( i.p.) of AMPH (1 mg/kg b.w.) for randomly chosen 20 days in 60 days of the chronic phase of the drug exposure with a subsequent blood collection (one hour after the last AMPH administration) and then exposed to the withdrawal from AMPH for 20 consecutive days and another blood sample collection, then the animals were sacrificed; the control SAL withdrawal group ( n = 10); control for the chronic phase of the drug exposure) – received the same procedure as the AMPH withdrawal group except for the repeated administration (i.p.) of 0.9% NaCl (SAL) instead of AMPH; the AMPH after withdrawal group (n = 10; AMPH challenge after withdrawal from the drug) was exposed to the same procedure as the AMPH withdrawal group with a subsequent single AMPH injection, then, next day at 09.00 a.m. blood samples were collected and the rats were sacrificed, and the control SAL after withdrawal group (n = 10; control for the drug challenge after the withdrawal phase) with the same procedure as within the AMPH challenge after withdrawal group however, with 20 injections of 0.9% NaCl (SAL) instead of AMPH during randomly chosen 20 days in 60 days of the chronic phase of SAL exposure.

The principles for the care and use of laboratory animals in research, as outlined by the Local Ethical Committee for the Care and Use of Laboratory Animals of the University of Science and Technology of Bydgoszcz, Poland, were strictly followed and all the protocols were reviewed and approved by the Committee (serial number 53/2012).

### Drug Administration

D-Amphetamine sulfate (Sigma, USA) was dissolved in sterile 0.9% NaCl (SAL) and administered (i.p.) by 20 injections at a dose of 1 mg/kg b.w. in a volume of 1 ml/kg. The control injections of SAL were performed by the same route and in the same volume. There is evidence to suggest that injections of AMPH given relatively far apart in time are more efficacious than those given more frequently (Robinson 1984; Robinson and Becker 1986) therefore, to obtain model of chronic, random AMPH treatment, we used the repeated intermittent twenty injections of a moderate dose of AMPH for 60 days. Injections of AMPH or SAL were administered for 20 randomly chosen days (one injection per randomly chosen day) in 60 days of the chronic AMPH/SAL treatment. Injections were carried out on the following days of the chronic AMPH/SAL treatment alone phase: 1^st^, 3^th^, 6^th^, 7^th^, 9^th^, 12^th^, 26^th^, 29^th^, 32^th^, 33^th^, 29^th^, 30^th^, 33^th^, 39^th^, 41^th^, 44^th^, 45^th^, 48^th^, 59^th^, 60^th^. All the injections were performed in the vivarium. Because exposure to high doses of AMPH results in hyperthermia and toxicities (Bowyer 2014; Camacho et al. 2019) which will enable the determination of the non-hyperthermic peripheral immune and endocrine response, the moderate dose of AMPH (1 mg/ml/kg b.w.) was chosen, based on the preliminary experiments and data reported by other authors (Swerdlow et al. 1993; Kubera et al. 2002; Ligeiro-Oliveira et al. 2004; Assis et al. 2006; Saito et al. 2008), concerning the effective reactivity to AMPH of the immune and neuroendocrine systems.

### Blood Collection

One hour after the last injection of the chronic AMPH treatment, or withdrawal from AMPH in the AMPH withdrawal group, or the next day after the last injection in the AMPH after withdrawal group, blood samples in a volume of 4 ml were collected by a cardiac puncture, under halothane anesthesia (Narkotane Zentiva, Prague, Czech Republic) according to the method described previously (Wrona et al. 2014).

### Total Blood Leukocyte and Leukocyte Subset Number

Total blood leukocyte and leukocyte subset numbers were determined according to the procedure that we previously described (Podlacha et al. 2016). Absolute blood leukocyte counts were determined with the hematology analyzer (Baker System 9120 CP, Biochem Immunosystems). Leukocyte subpopulations (lymphocytes, monocytes, granulocytes) were assessed by microscopic examination of the peripheral blood suspension smears stained in a centrifuge (Aerospray Slide Stainer, 7120 Wescor, USA) according to the May-Grünwald and Giemsa methods. The slides were inspected under oil immersion with a light microscope. Two smears of blood sample were prepared and percentage of lymphocytes, monocytes and granulocytes were assessed on 200 cells per smear. The absolute number of each leukocyte subset was calculated as an absolute leukocyte number x percentage of the individual leukocyte subset.

### Cytometric Analysis of the Lymphocyte Populations and Subpopulations

**Fig. 2:**
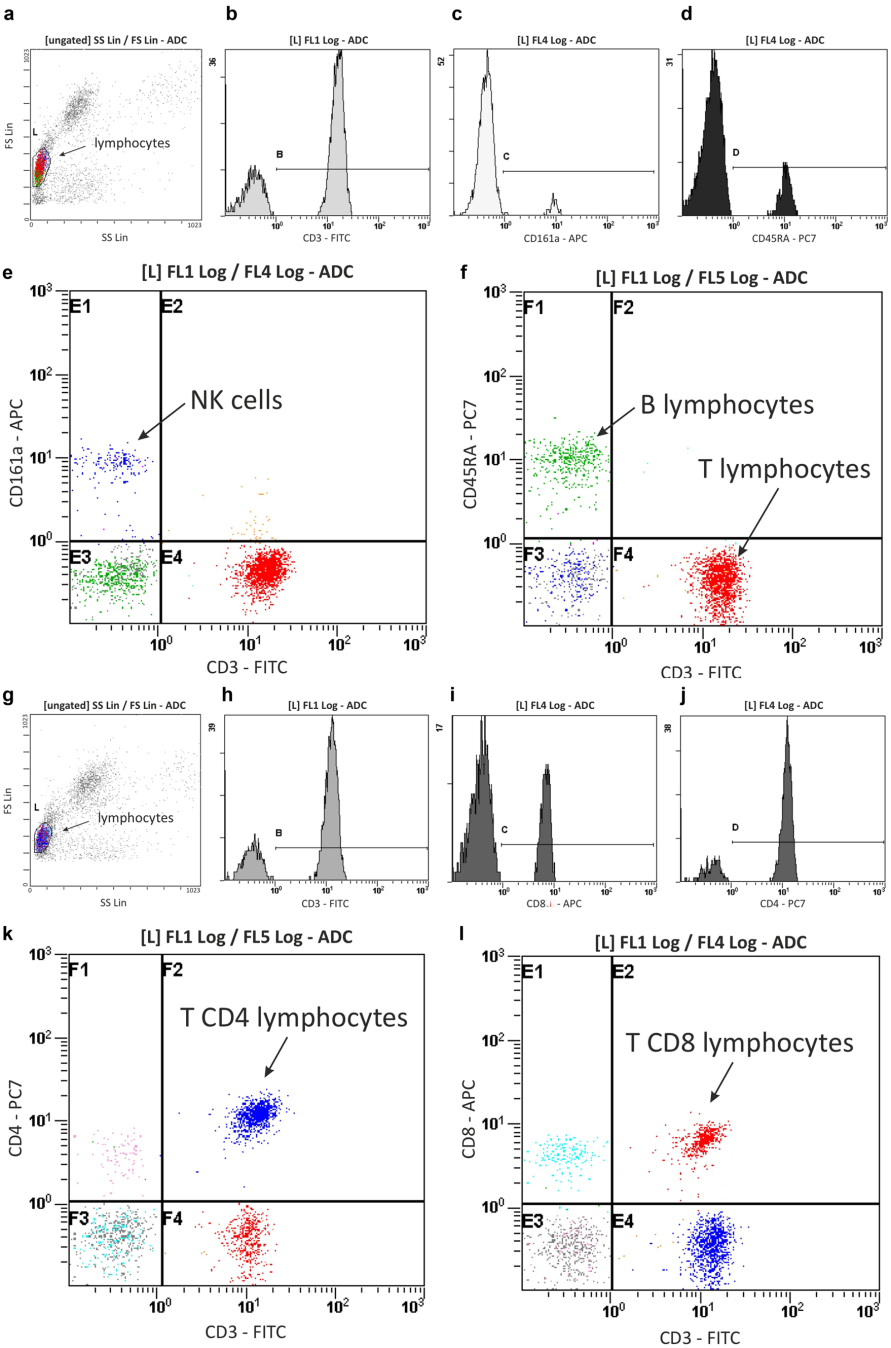
Representative flow cytometry graphs showing the cell surface marker analysis of peripheral blood lymphocyte populations (T, B, NK) and subsets of T lymphocytes (TCD4^+^, TCD8^+^). Explanations: a and g graphs: FS vs. SS plot of the whole blood with lymphocytes gated for analysis of T, B and NK lymphocytes (a), as well as TCD4^+^ and TCD8^+^ (g) lymphocyte subsets, circle highlights the lymphocyte population; b-d and h-j graphs show identification of different lymphocyte populations and T lymphocyte subsets based on the surface marker expression on lymphocytes. Histograms present the cell count (Y-axis) and fluorescent intensity (X-axis), unstained control cells (left) and cells stained (right) with antibody against the surface protein on lymphocytes: CD3-FITC (b and h graphs), CD161a-APC (c graph), CD45RA-PC7(d graph), CD8-APC (i graph) and CD4-PC7 (j graph); e–f and k-l graphs show identification of different lymphocyte populations and T lymphocyte subsets based on the surface marker expression on lymphocytes. Cytograms present quadrant regions with analyzed lymphocyte populations and T lymphocyte subsets: NK cells (CD3^−^ CD161a^+^) (e graph), B lymphocytes (CD3^−^ CD45RA^+^) and T lymphocytes (CD3^+^ CD45RA^−^) (f graph) as well as T CD4^+^ lymphocytes (CD3^+^ CD4^+^) (k graph) and T CD8^+^ lymphocytes (CD3^+^ CD8^+^) (l graph); FITC—fluorescein isothiocyanate; PC7—phycoerythrin cyanin 7; APC—allophycocyanin

A three-color combination of fluorescent monoclonal antibodies was used in the study to identify T (CD3^+^), B (CD45RA^+^), NK (CD161a^+^) lymphocytes and T lymphocyte subsets of the T helper (CD3^+^CD4^+^) and T cytotoxic (CD3^+^CD8^+^) lymphocyte percentage according to the method that we previously described (Listowska et al. 2015; Grembecka et al. 2020) with some modifications. Twenty five µl of the whole blood was added to 25 µl of IOTest CD3-FITC/CD45RA-PC7/CD161a-APC or CD3-FITC/CD4-PC7/CD8-APC (Beckman Coulter, USA) according to the manufacturer’s instructions. Erythrocytes were lysed (Versalyse, Beckman Coulter) and then samples were mixed and incubated at room temperature for 20 min in darkness. After incubation, 25 μl of Fixative Solution (Beckman Coulter, USA) and 700 μl of PBS was added to the separate sample. Samples were protected from light and stored at 4 °C until flow cytometry had been performed with a Cytomics FC 500 flow cytometer (Beckman Coulter, USA) and MXP Software. The percentages of lymphocyte population and T lymphocyte subsets were assessed during the assay, gaiting on forward and side scatter characteristics (Fig. 2). Then, absolute numbers of the lymphocyte population and their subsets were calculated on respective counts of the absolute number of leukocytes and a percentage of the T, B, NK, CD4^+^T and CD8^+^T lymphocytes.

### Determination of Peripheral Blood-derived Production of the Pro-inflammatory Interferon-γ (IFN-γ) and Anti-inflammatory Interleukin (IL)-4

Peripheral blood mononuclear cells (PBMC) were examined for their ability to produce pro-inflammatory IFN-γ and anti-inflammatory IL-4 in response to concanavalin A (Con-A) stimulation according to the procedure described previously (Wrona et al. 2014). Briefly, PBMC suspension in RPMI-1640 with a 10% calf bovine serum were seeded at a concentration of 4 × 10^6^ cells/ml in 24-well Corning tissues culture plates, and then stimulated with a Con-A solution (2.5 μg/ml) or remained non-stimulated (control). The PBMC were incubated in 37 °C. Cell-free supernatants were collected 48 h later and stored at -80 °C until assayed.

The IFN-γ and IL-4 concentrations in the supernatants were determined by enzyme-linked immunoassay (ELISA) using a commercially available kit (Rat-IFN-γ and Rat-IL-4 ELISA kits R&D, USA) according to the manufacture’s instructions and our previous study (Wrona et al. 2014). Briefly, 50 µl of standards or samples were dispensed into 96 wells coated with rat IFN-γ and IL-4 antibody, respectively, and incubated for 2 h (IFN-γ) or 1 h (IL-4) at room temperature. After extensive washing, 100 µl of the biotinylated anti-IFN-γ or anti-IL-4 were added to each well, and the plates were incubated for 30 min (IFN-γ) or 1 h (IL-4) at room temperature. The wells were again washed 3 times, 100 µl of Streptavidin-HRP was added and incubation was carried out for 30 min. 3,3´,5,5´-tetramethylbenzidine (TMB) (100 μl/well) was used as the chromogen for the colorimetric assay. The reaction was stopped after 10 min by adding a 100 µl/well of stop solution and the absorbance was determined using the DTX 880 Multimode Detector (Beckman Coulter, USA) system set to 450 nm. Cytokine concentrations were calculated based on the standard curve generated by Beckman Coulter´s Biomek software program, based on the absorbance of standard samples. The sensitivity of detection was 2 pg/ml for IFN-γ and 3 pg/ml for IL-4.

### Determination of Plasma Corticosterone Concentration

The plasma corticosterone concentration was measured by radioimmunoassay using a commercially available kit (rCorticosterone [^125^I RIA KIT, isotop Institute of Isotopes Co, LTD, Budapest, Hungary] and Wizard 1470 gamma counter (Pharmacia – LKB, Turku, Finland). The measures were made in a duplicate. The sensitivity of the assay was 0.01 ng/tube.

### Statistical Analysis

The data is presented as mean ± SE. The normality of the distribution of the variables was checked with Kolmogorow-Smirnov test and the homogeneity of the variances with a Levene test. As the outcome of Kołmogorow-Smirnov test indicated that all data, except for plasma corticosterone concentration, was distributed normally, we used for further statistical evaluation of immune parameters a two-way ANOVA with the following factors: treatment (AMPH withdrawal, AMPH after withdrawal, SAL withdrawal, SAL after withdrawal) and time point of the experiment (chronic AMPH treatment alone, addictive phase), withdrawal from AMPH or SAL, and AMPH or SAL challenge after withdrawal). The differences in means were further analyzed with Tukey’s HSD post hock test. As the outcome of the Kolmogorow-Smirnov test for the plasma corticosterone concentration indicated that the data was not distributed normally, we used non-parametric tests for further analysis. Plasma corticosterone concentration data was evaluated using the Kruskal–Wallis one way ANOVA or Mann–Whitney U test. A p value equal to or lower than 0.05 was consider statistically significant.

## Results

### Total Blood and Spleen Leukocyte Number, Spleen and Body Weight

The total blood and spleen leukocyte number is presented in Table 1. Two-way ANOVA revealed a significant effect of both factors (Table 2) on the total blood (p ≤ 0.001 and p ≤ 0.01, respectively) and spleen (p ≤ 0.01 and p ≤ 0.05, respectively) leukocyte number. In comparison with the control SAL and AMPH withdrawal groups, there was a significant decrease in the total blood leukocyte number after the AMPH chronic treatment alone and AMPH challenge after withdrawal (Tukey’s post hoc test). In addition, a significantly lower level of the total blood (p ≤ 0.001) and spleen (p ≤ 0.05) leukocyte numbers in the AMPH after withdrawal group, as compared to the AMPH withdrawal group, was observed.

**Table 1:** The total blood and spleen leukocyte number, blood TCD4^+^/TCD8^+^ lymphocyte ratio, spleen weight, relative spleen weight and body weight in rats subjected to chronic, random injections (20 injections in 60 days, i.p.) of amphetamine (AMPH chronic) or saline (SAL chronic), followed by a withdrawal of AMPH (n = 10) or SAL (n = 10) for 20 consecutive days (AMPH/SAL withdrawal) and subsequent single injections of AMPH (AMPH after withdrawal, n = 10) or SAL (SAL after withdrawal, n = 10)

Parameters/Phase	AMPH chronic	AMPH withdrawal	AMPH after withdrawal	SAL chronic	SAL withdrawal	SAL after withdrawal
blood leukocyte number (No./μl)	6539.4 ± 1549.8 ^***^	8650.8 ± 718.1 ^$$^	4625.6 ± 872.7 ^***; $$; ###^	9928.1 ± 628.0	9277.2 ± 627.7	9615.0 ± 870.6
blood TCD4^+^/TCD8^+^ cell ratio	1.5 ± 0.4	2.1 ± 0.9	2.0 ± 0.7	1.8 ± 0.6	2.2 ± 0.9	2.2 ± 0.9
spleen leukocyte number (No./μl)		252600.0 ± 29098.5	142560.0 ± 26831.5 ^#^		171600.0 ± 62154.3	102000.0 ± 47532.7
spleen weight (mg)		780.0 ± 90.0	730.0 ± 60.0		800.0 ± 130.0	790.0 ± 140.0
relative spleen weight (mg/100 g b.w.)		189.3 ± 15.5	161.68 ± 14.0^#^		199.2 ± 39.7	169.7 ± 23.86
body weight (g):						
initial	344.5 ± 36.7	349.4 ± 32.0	345.6 ± 42.3	357.0 ± 49.4	342.0 ± 58.0	372.0 ± 38.2
after treatment	432.9 ± 39.5 ^&&&^	413.3 ± 42.6	452.5 ± 25.5 ^&&&^	445.0 ± 37.1 ^&&&^	427.0 ± 37.0 ^&^	463.0 ± 30.3 ^&^

Data is presented as a mean ± SD

^***^p ≤ 0.001- significance of differences in comparison to saline treated controls (SAL); $$p ≤ 0.01–significance of differences in comparison to AMPH chronic; #p ≤ 0.05, ### p ≤ 0.001–significance of differences in comparison to AMPH withdrawal;&p ≤ 0.05, &&&p ≤ 0.001- significance of differences in comparison to the initial body weight Tukey post hoc after two-way ANOVA

**Table 2:** The results of two-way ANOVA for peripheral blood and spleen immune parameters, body and spleen weight with the following factors: treatment (AMPH/SAL) and phase of AMPH/SAL challenge (AMPH/SAL chronic, AMPH/SAL withdrawal, AMPH/SAL after withdrawal) in rats subjected to chronic, random injections (20 injections in 60 days, i.p.) of amphetamine (AMPH chronic) or saline (SAL chronic), followed by a withdrawal of AMPH (n = 10) or SAL (n = 10) for 20 consecutive days (AMPH/SAL withdrawal) and subsequent single injections of AMPH (AMPH after withdrawal, n = 10) or SAL (SAL after withdrawal, n = 10)

Parameters/Factors	Two-way ANOVA		
	Phase	Treatment	Interaction
	df(1,10)	df(1,10)	df(1,10)
Leukocytes (spleen)	F=16.0 ^#^	F=7.32 ^&^	F=0.81
	df(2,34)	df(1,34)	df(2,34)
Leukocytes (blood)	F=10.72 ^*^	F=91.23 ^*^	F=14.83 ^*^
	df(2,34)	df(1,34)	df(2,34)
Granulocytes	F=6.23 ^#^	F=33.59 ^*^	F=4.38 ^&^
Monocytes	F=2.58^&^	F=3.45^&^	F=4.15 ^&^
Lymphocytes	F=8.22 ^#^	F=75.94 ^*^	F=13.47 ^*^
	df(2,33)	df(1,33)	df(2,33)
T lymphocytes	F=13.74 ^*^	F=62.66 ^*^	F=16.37 ^*^
	df(2,32)	df(1,32)	df(2,32)
B lymphocytes	F=4.06 ^&^	F=47.56 ^*^	F=4.31 ^&^
	df(2,31)	df(1,31)	df(2,31)
Natural killer cells	F=19.76 ^*^	F=2.56	F=17.74 ^*^
	df(2,34)	df(1,34)	df(2,34)
TCD4^+^ lymphocytes	F=11.212 ^*^	F=44.324 ^*^	F=12.0 ^*^
TCD8^+^ lymphocytes	F=5.95 ^#^	F=15.81 ^#^	F=5.55 ^#^
TCD4^+^/TCD8^+^ cell ratio	F=2.06	F=0.6	F=0.06
	df(2,34)	df(1,34)	df(2,34)
Interferon-γ	F=28.27 ^*^	F=87.84 ^*^	F=24.02 ^*^
	df(2,31)	df(1,31)	df(2,31)
Interleukin-4	F=58.92 ^*^	F=104.12 ^*^	F=58.56 ^*^
	df(2,22)	df(1,22)	df(2,22)
Relative spleen weight	F=9.49 ^#^	F=0.94	F=0.011
	df(2,150)	df(1,150)	df(2,150)
Body weight	F=5.10 ^#^	F=4.63 ^&^	F=0.32

Data is presented as F value, df – number of degrees of freedom; & p ≤ 0.05; # p ≤ 0.01; * p ≤ 0.001

There was a significant effect of the phase of the experiment and no significant effect of the treatment and factors’s interaction on the spleen and body weight (Table 1, Table 2). However, the results of two-way ANOVA revealed a significant effect (p ≤ 0.01) of the phase of the experiment on the relative spleen weight. A Tukey’s post hoc test showed a significantly (p ≤ 0.01) lower the relative spleen weight in the AMPH after withdrawal from the drug rather than the AMPH withdrawal group (Table 1).

### Total Blood Granulocyte, Monocyte, Lymphocyte and Lymphocyte Subpopulation Number

**Fig. 3:**
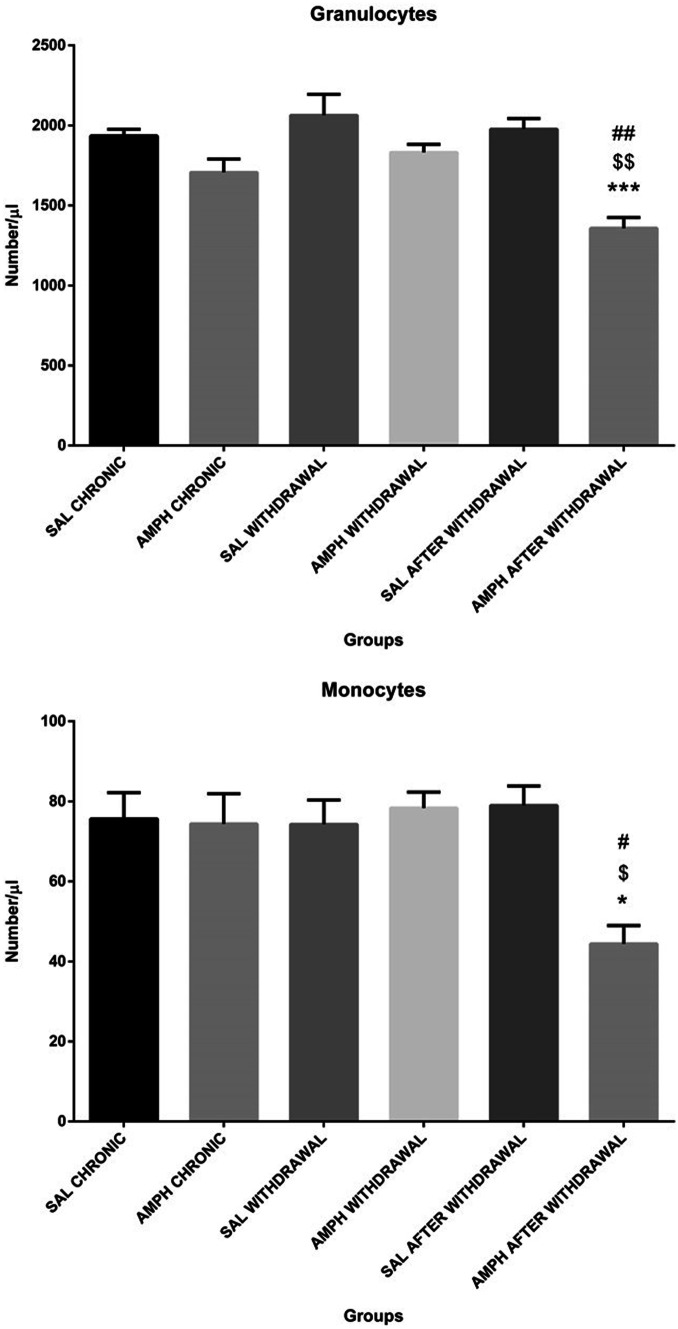
The total number of granulocytes (top) and monocytes (bottom) in the peripheral blood in rats subjected to chronic, random injections (20 injections in 60 days, i.p.) of amphetamine (AMPH chronic) or saline (SAL chronic), followed by a withdrawal of AMPH (n = 10) or SAL (n = 10) for 20 consecutive days (AMPH/SAL withdrawal) and subsequent single injections of AMPH (AMPH after withdrawal, n = 10) or SAL (SAL after withdrawal, n = 10). Explanations: * p ≤ 0.05:0.05 ,*** p ≤ 0.001–significance of differences in comparison to saline treated controls (SAL); $ p ≤ 0.05, $$ p ≤ 0.01significance of differences in comparison to AMPH chronic; # p ≤ 0.05, ## p ≤ .001- significance of differences in comparison to AMPH withdrawal; Tukey post hoc after two-way ANOVA

Two-way ANOVA showed that the phase of the experiment (p ≤ 0.01 and p ≤ 0.05) and treatment (p ≤ 0.001 and p ≤ 0.05 for granulocytes and monocytes, respectively) and their interactions (p ≤ 0.05) significantly influenced the total blood granulocyte and monocyte numbers (Table 2). As shown in Fig. 3, significantly lower total blood granulocyte and monocyte numbers were observed in the AMPH after withdrawal group in comparison with the respective controls (granulocytes: p ≤ 0.001; monocytes: p ≤ 0.05), AMPH chronic treatment alone (granulocytes: p ≤ 0.01; monocytes: p ≤ 0.05) and AMPH withdrawal group (granulocytes: p ≤ 0.01; monocytes: p ≤ 0.05) values (Tukey’s post hoc test).

**Fig. 4:**
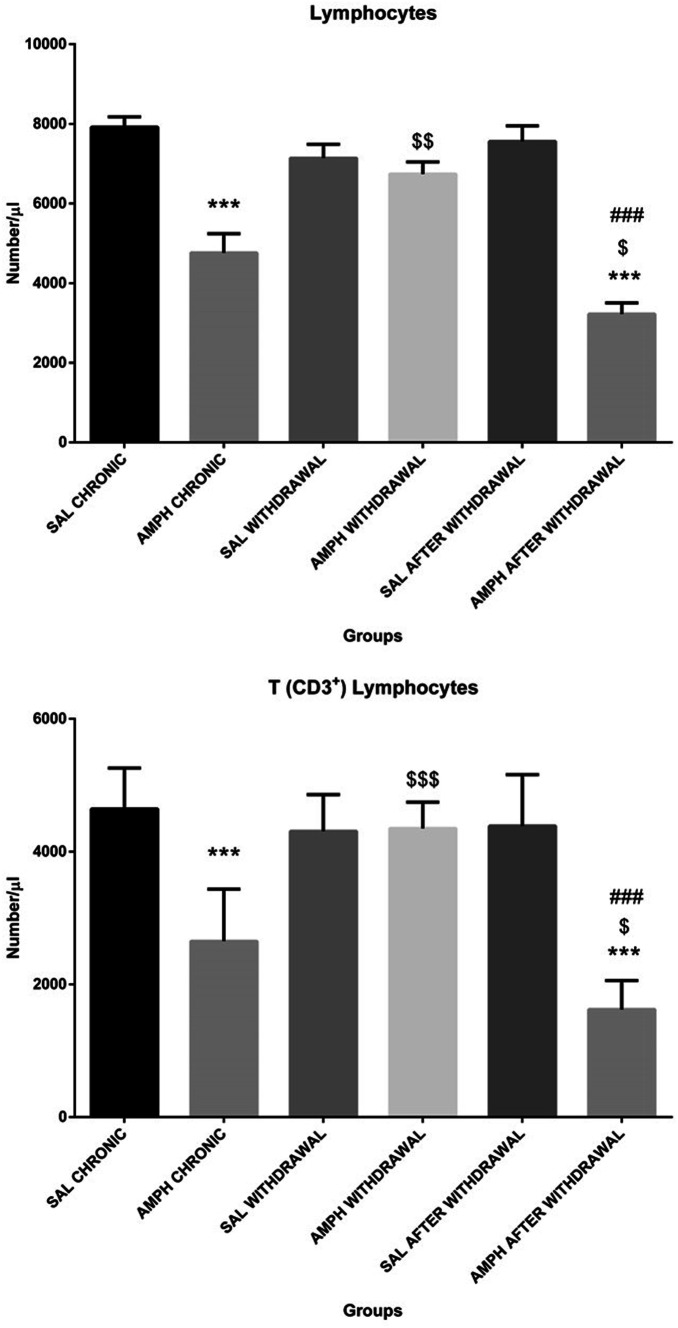
The total number of lymphocytes (top) and T lymphocytes (bottom) in the peripheral blood in rats subjected to chronic random injections (20 injections in 60 days, i.p.) of amphetamine (AMPH chronic) or saline (SAL chronic), followed by a withdrawal of AMPH (n = 10) or SAL (n = 10) for 20 consecutive days (AMPH/SAL withdrawal) and subsequent single injections of AMPH (AMPH after withdrawal, n = 10) or SAL (SAL withdrawal, n = 10). Explanations: ***p ≤ 0.001–significance of differences in comparison to saline treated controls (SAL); $ p ≤ 0.05, $$ p ≤ 0.01, $$$ p ≤ 0.001–significance of differences in comparison to AMPH chronic; ### p ≤0.001- significance of differences in comparison to AMPH withdrawal; Tukey post hoc after two-way ANOVA

The results of two-way ANOVA showed a significant effect of both factors (p ≤ 0.01 and p ≤ 0.001 for the phase of the experiment and treatment, respectively) and their interactions (p ≤ 0.001) on the total blood lymphocyte number (Table 2). Post hoc test revealed a significantly (p ≤ 0.001) lower the total blood lymphocyte number after chronic, random injections of AMPH alone and after AMPH challenge followed by the withdrawal, in comparison with the respective control SAL values (Fig. 4). There was a significantly lower number of blood lymphocytes within the AMPH after withdrawal group in comparison with the AMPH chronic treatment alone (p ≤ 0.05) and AMPH withdrawal group (p ≤ 0.001). Moreover, after the withdrawal from AMPH phase, the blood lymphocyte number returned to the control value.

**Fig. 5:**
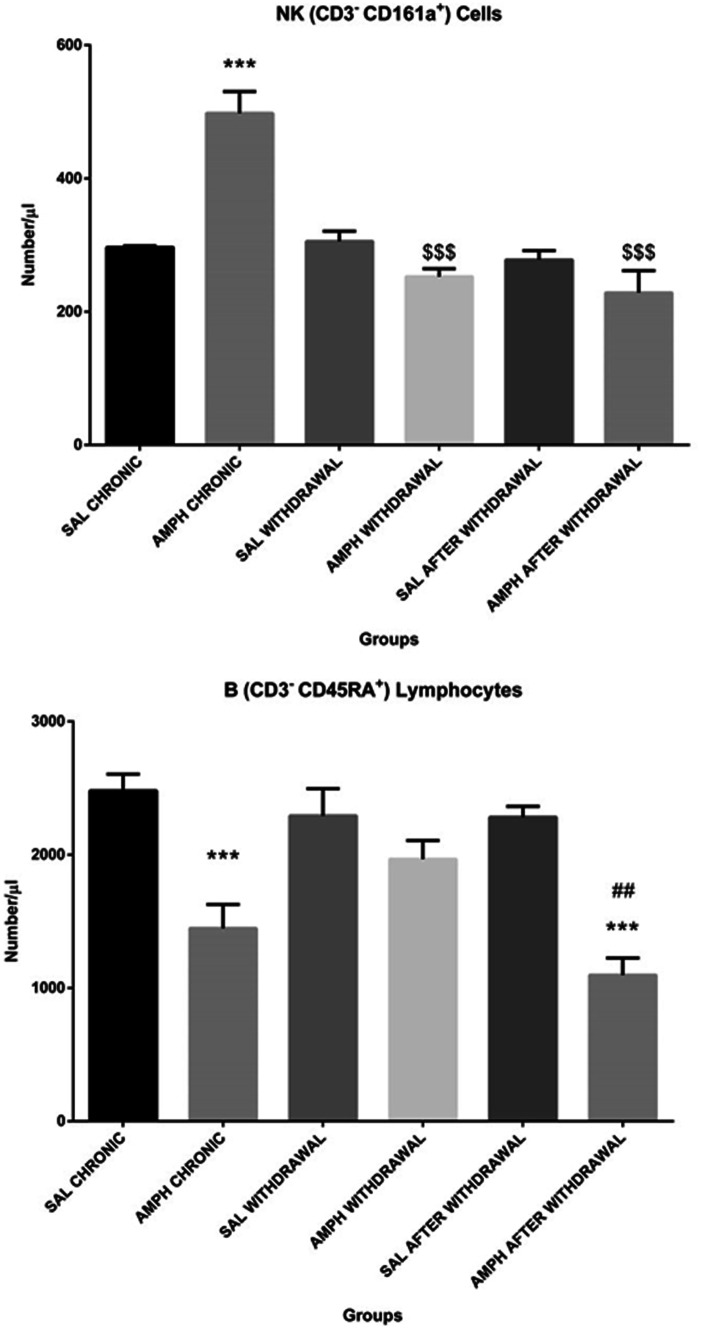
The total number of natural killer (NK) cells (top) and B lymphocytes (bottom) in the peripheral blood in rats subjected to chronic random injections (20 injections in 60 days, i.p.) of amphetamine (AMPH chronic) or saline (SAL chronic), followed by a withdrawal of AMPH (n = 10) or SAL (n = 10) for 20 consecutive days (AMPH/SAL withdrawal) and subsequent single injections of AMPH (AMPH after withdrawal, n = 10) or SAL (SAL after withdrawal, n = 10). Explanations: ***p ≤ 0.001–significance of differences in comparison to saline treated controls (SAL); $$$ p ≤ 0.001–significance of differences in comparison to AMPH chronic; ## p ≤0.01- significance of differences in comparison to AMPH withdrawal; Tukey post hoc after two-way ANOVA

As shown in Table 2, there was a significant effect of both factors and their interaction on lymphocyte subpopulations determined by flow cytometric immunophenotyping except for the treatment factor for natural killer cells. There was a significant effect of the phase of the experiment, treatment and interaction on T (CD3^+^) lymphocytes (p ≤ 0.001), B (CD3^−^CD45RA^+^) lymphocytes (p ≤ 0.05) and p ≤ 0.001, respectively), NK (CD3^−^CD161a^+^) cells (p ≤ 0.001 for the phase of the experiment and the interaction), TCD4^+^ (TCD3^+^CD4^+^CD8^−^) lymphocytes (p ≤ 0.001), TCD8^+^ (TCD3^+^CD4^−^CD8^+^) lymphocytes (p ≤ 0.01). However, there was no significant effect of either factors or their interaction on blood TCD4^+^/TCD8^+^ lymphocyte ratio. Tukey post hoc test showed a significantly decreased total T (CD3^+^) and B (CD3^−^CD45RA^+^) lymphocyte numbers after AMPH chronic treatment alone and in the AMPH after withdrawal group in comparison with the control SAL group (Figs. 4 and 5, p ≤ 0.001). A significantly lower T (CD3^+^) lymphocyte number in the AMPH after withdrawal group, rather than AMPH chronic treatment alone, was noted. In contrast, the total number of NK (CD3^−^CD161a^+^) cells significantly (p ≤ 0.001) increased after the chronic, random AMPH treatment alone, in comparison with the respective controls, and returned to the control values in the AMPH withdrawal and AMPH after withdrawal groups (Fig. 5).

**Fig. 6:**
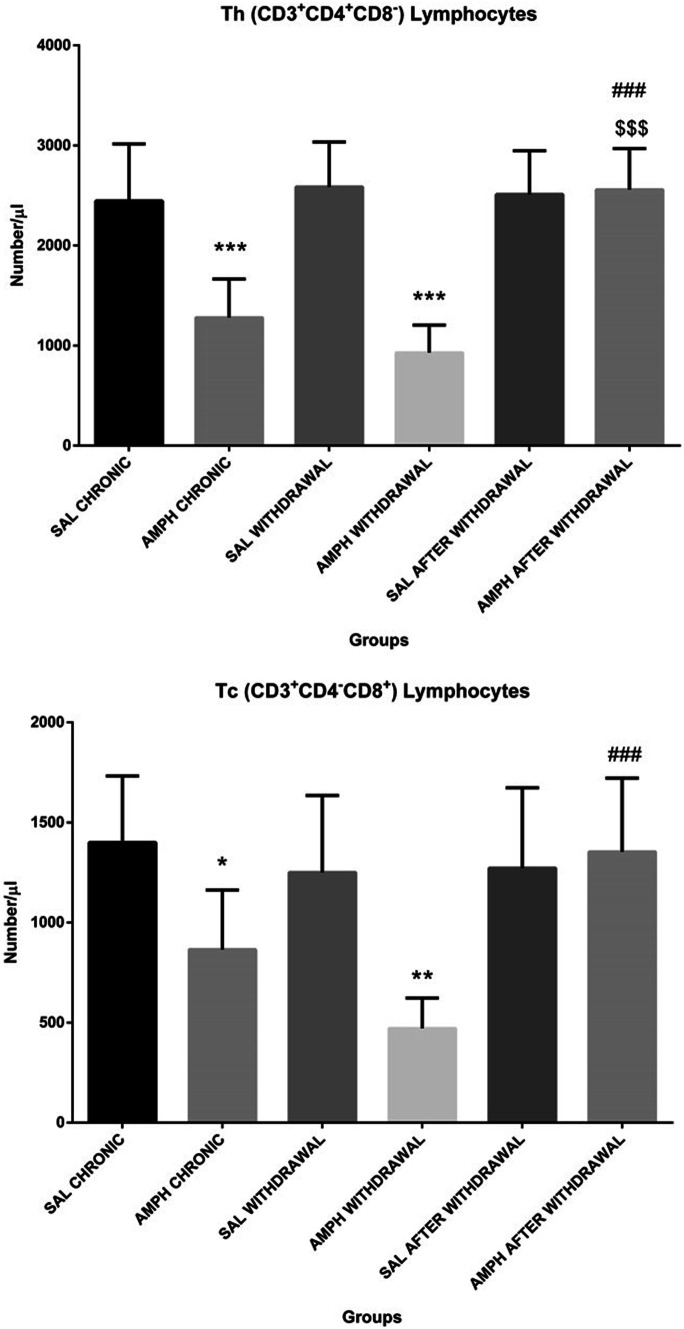
The total number of TCD4^+^ (top) and TCD8^+^ lymphocytes (bottom) in the peripheral blood in rats subjected to chronic random injections (20 injections for 60 days, one injection per day, i.p.) of amphetamine (AMPH chronic) or saline (SAL chronic), followed by a withdrawal of AMPH (n = 10) or SAL (n = 10) for 20 consecutive days (AMPH/SAL withdrawal) and subsequent single injections of AMPH (AMPH relapse, n = 10) or SAL (SAL relapse, n = 10). Explanations: * p ≤ 0.05, ** p ≤ 0.01, ***p ≤ 0.001–significance of differences in comparison to saline treated controls (SAL); $$$ p ≤ 0.001–significance of differences in comparison to AMPH chronic; ### p ≤0.001- significance of differences in comparison to AMPH withdrawal; Tukey post hoc after two-way ANOVA

Figure 6. presents the total number of blood TCD4^+^ (TCD3^+^CD4^+^CD8^−^, at the top) and TCD8^+^ (TCD3^+^CD4^−^CD8^+^ at the bottom) lymphocytes after the chronic AMPH/SAL treatment alone, in the AMPH/SAL withdrawal and AMPH/SAL after withdrawal groups. In comparison with the control SAL chronic treatment alone and withdrawal, there were significant decreases in both TCD4^+^ (p ≤ 0.001) and TCD8^+^ (p ≤ 0.05 vs. SAL chronic; p ≤ 0.01 vs. SAL withdrawal) lymphocyte numbers. In contrast, after AMPH challenge followed by the withdrawal, the TCD4^+^ and TCD8^+^ lymphocyte numbers returned to the control value and were significantly (p ≤ 0.001) higher than after the chronic AMPH treatment alone and the AMPH withdrawal groups. Moreover, there were no significant changes in the TCD4^+^/TCD8^+^ cell ratio in the AMPH treated groups.

### Production of Blood Pro-inflammatory Cytokine Interferon-ɤ (IFN-y and Anti-inflammatory Cytokine Interleukin-4 (IL-4)

**Fig. 7:**
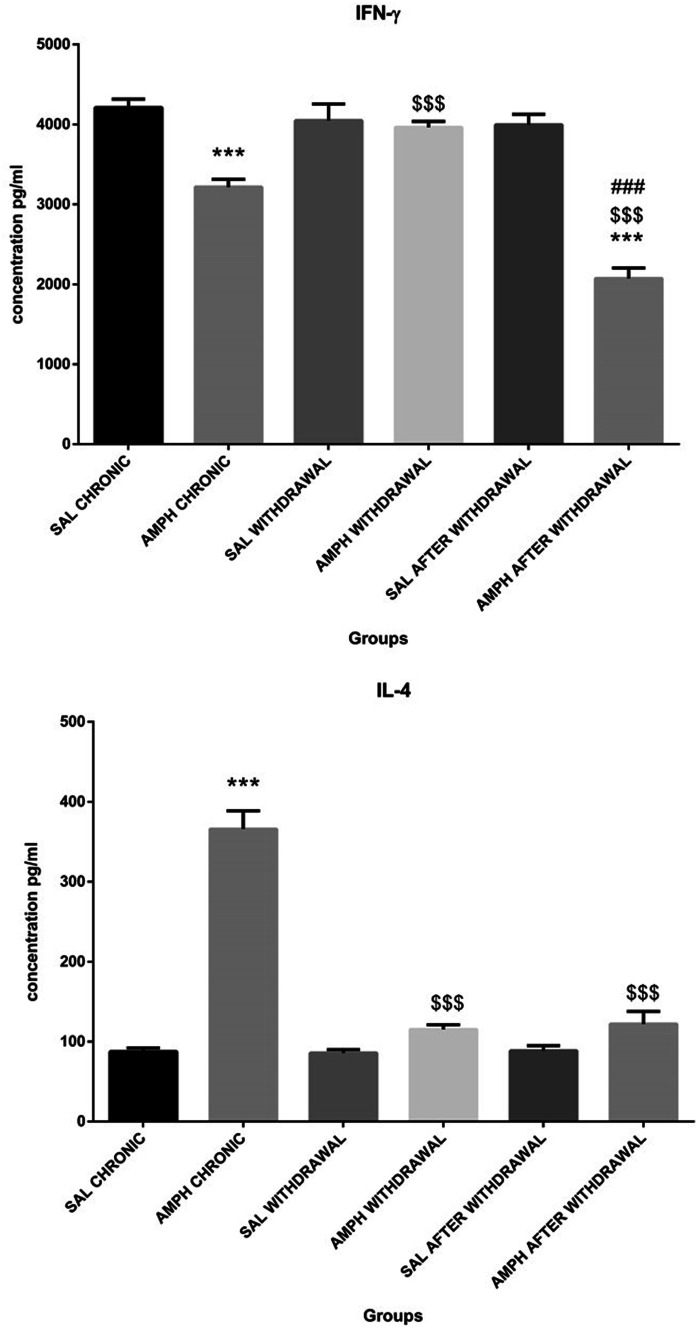
Production (concentration in supernatants) of pro-inflammatory interferon gamma (IFN-γ) (top) and anti-inflammatory interleukin 4 (IL-4) by peripheral blood mononuclear cells in response to concanavalin-A (Con-A) stimulation in rats subjected to chronic, random injections (20 injections in 60 days, i.p.) of amphetamine (AMPH chronic) or saline (SAL chronic), followed by a withdrawal of AMPH (n = 10) or SAL (n = 10) for 20 consecutive days (AMPH/SAL withdrawal) and subsequent single injections of AMPH (AMPH after withdrawal, n = 10) or SAL (SAL after withdrawal, n = 10). Explanations: ***p ≤ 0.001–significance of differences in comparison to saline treated controls (SAL); $$$ p ≤ 0.001- significance of differences in comparison to AMPH chronic; ### p ≤ .0.001- significance of differences in comparison to AMPH withdrawal; Tukey post hoc after two-way ANOVA

Results of two-way ANOVA for the factors effects and their interaction are presented in Table1. There was a significant effect of both factors and their interaction (p ≤ 0.001) on IFN-ɤ and IL-4 production by blood concanavalin-A-stimulated lymphocytes. As shown in Fig. 7, production of IFN-ɤ was significantly lower after the chronic treatment with AMPH alone and in the AMPH after withdrawal group, in comparison with the control SAL chronic and SAL after withdrawal values. In the AMPH after withdrawal group, blood lymphocyte ability to produce IFN-ɤ was significantly (p ≤ 0.001) lower than in the chronic AMPH treatment alone and the AMPH withdrawal groups. In the AMPH withdrawal group, Con-A-stimulated production of IFN-ɤ returned to the control value. In contrast, production of IL-4 after chronic, random injections of AMPH, significantly (p ≤ 0.001) increased in comparison with the control SAL chronic group (Fig. 7). In the AMPH withdrawal and AMPH after withdrawal groups, blood production of IL-4 was not significantly different from the respective control groups.

### Plasma Corticosterone Concentration

**Fig. 8:**
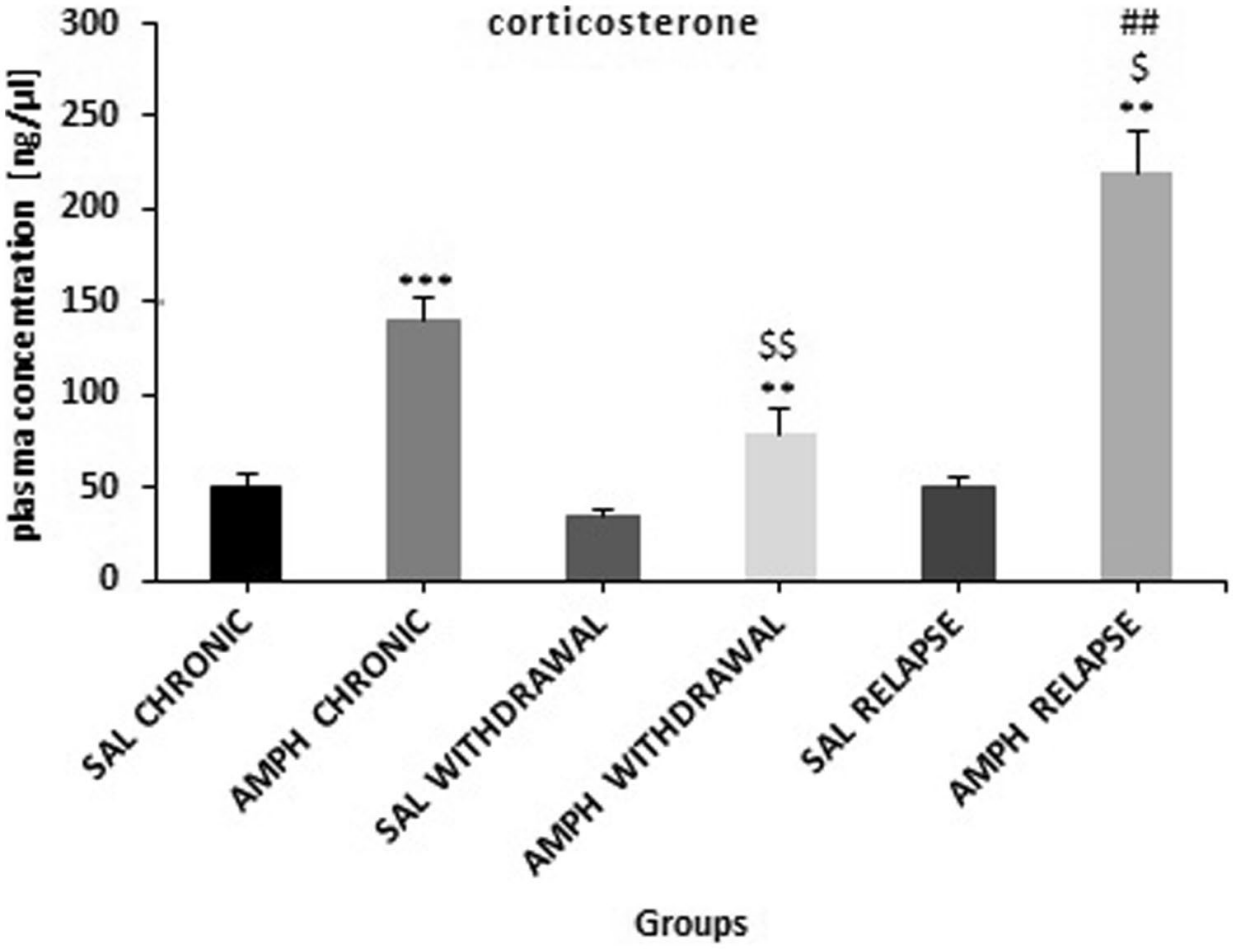
The plasma corticosterone concentration in rats subjected to chronic, random injections (20 injections in 60 days, i.p.) of amphetamine (AMPH chronic) or saline (SAL chronic), followed by a withdrawal of AMPH (n = 10) or SAL (n = 10) for 20 consecutive days (AMPH/SAL withdrawal) and subsequent single injections of AMPH (AMPH after withdrawal, n = 10) or SAL (SAL after withdrawal, n = 10). Explanations: ** p ≤ 0.01, ***p ≤ 0.001–significance of differences in comparison to saline treated controls (SAL); $ p ≤ 0.05, $$ p ≤ 0.01–significance of differences in comparison to AMPH chronic; ## p ≤ 0.01- significance of differences in comparison to AMPH withdrawal; Kruskal–Wallis one way ANOVA or Mann–Whitney U test

Kruskal–Wallis one way ANOVA revealed significant (p ≤ 0.001) differences in the plasma corticosterone concentration during the experiment. As shown in Fig. 8, in comparison with the control SAL groups, there was a significant increase in the plasma corticosterone concentration in the AMPH chronic treatment alone (p ≤ 0.001), AMPH withdrawal (p ≤ 0.01) and AMPH after withdrawal (p ≤ 0.01) groups. The plasma corticosterone concentration was significantly higher after challenge of the single injection of AMPH after withdrawal, when compared to the AMPH chronic (p ≤ 0.05) and AMPH withdrawal (p ≤ 0.01) groups.

## Discussion

As far as we know, this is the first report on long-term consequences of the chronic, random AMPH treatment of a low dose (1 mg/kg. b.w.), withdrawal from the AMPH treatment, and an acute exposure to AMPH after the withdrawal on the peripheral immune and corticosterone responses in rats. The present study shows that the chronic, random AMPH treatment and the acute challenge of AMPH after the long-term withdrawal phase, were capable of significantly suppressing the peripheral inflammatory response, which clearly indicates the immune system activation in response to the drug exposure. The major findings of this study are that single injections of AMPH after withdrawal from the drug, produced more accelerated immunosuppressive effects than the chronic phase of the random drug treatment alone. This was manifested as progressive lower levels of the immune parameters, the lack of immunostimulative effects, an augmented response of the hypothalamic–pituitary–adrenal (HPA) axis, as indicated by the highest plasma corticosterone concentration, in rats exposed to the acute AMPH treatment after the withdrawal. It suggests that chronic, random AMPH treatment acts as a stressor and sensitizes immunosuppressive effects and the stress response, supporting opinion that sensitized animals show exaggerated response when subsequently challenged with an injection of AMPH or further stress (Antelman et al. 1980; Robinson and Becker 1986).

In fact, there is no data on long-term effects of a chronic, random AMPH treatment with withdrawal and a subsequent challenge of the drug after the withdrawal phase on the peripheral immune status in rodents. However, decreased numbers of all lymphocyte populations were observed after chronic, regular AMPH exposure alone (Freire-Garabal et al. 1991; Basso et al. 1999; Llorente-Garcia et al. 2009). According to Camacho et al. (2019), AMPH reduced, in particular, T lymphocyte number in the blood and this decrease was probably due to T-cells leaving the circulation and down regulation of their genes. Chang et al. (2010) reported that cocaine- and amphetamine-regulated transcript treatment in male C57BL/6 mice subjected to ischemic stroke reduced blood TCD4^+^/TCD8^+^ ratio and pro-inflammatory cytokine expression. Our previous study showed that repeated administration of AMPH in a moderate dose of 1 mg/kg per day (i.p.) for 5 consecutive days produced a marked decrease in the total number of TCD4^+^ cells and remained without a significant effect on TCD8^+^ cell number in the blood. In contrast to the decreased TCD4^+^/TCD8^+^ ratio in the blood, in the spleen the total TCD8^+^ lymphocyte number was decreased thus an increased TCD4^+^/TCD8^+^ ratio was observed in the spleen (data not shown). Moreover, there are a few studies that document the effects of methamphetamine (METH), on T cells. According to Mata et al. (2015), METH decreases TCD4^+^ cell whereas increases TCD8^+^ cell frequency and alters pro-inflammatory cytokine production in a model of drug abuse. In mice, chronic METH administration reduces the number of TCD4^+^ and TCD8^+^ lymphocytes in the spleen (Harms et al. 2012) suggesting that METH contributes to T cell function and migration. Pacifici et al. (1999, 2001) revealed decreased percentage of T and TCD4^+^ lymphocytes following exposure to Ecstasy (MDMA) in humans.

As far as chronic, random AMPH injection effects are concerned, our results are consistent with the mentioned reports and show signs of both the prolonged AMPH exposure alone and an acute AMPH challenge after the withdrawal adversely affecting the T- and B-cell numbers, in particular, TCD4^+^ and TCD8^+^ lymphocytes. It suggests that lymphopenia could be due to loss of these lymphocytes. It should be pointed out, that decreases in leukocyte and lymphocyte numbers, except for TCD4^+^ and TCD8^+^ subsets, were even more pronounced in response to the single injection of the drug after the withdrawal phase rather than after the AMPH treatment alone, whereas the decreased numbers of TCD4^+^ and TCD8^+^ lymphocytes, concomitantly with the recovered numbers of other white blood cells, were observed even in the late phase of the withdrawal. Moreover, in parallel to AMPH-induced immunosuppressive effects, a significantly lower the relative spleen weight in the AMPH after withdrawal rather than AMPH withdrawal group was observed in the present study. Others also reported that chronic, regular or acute administration of AMPH to mice reduced the thymus and spleen weight, decreased the proliferative activity of splenocytes in response to Con-A administration (Kubera et al. (2002), and weakened the resistance to bacteria, candidiasis and tumors (Wemm and Sinha 2019). Ours results are in agreement with those observations that chronic AMPH treatment facilitates immunosuppression (Schloesser et al. 2009) in response to a novel aversive stimulus (Basso et al. 1999) and induces sensitization of HPA axis to a subsequent stressor (Barr et al. 2002) thus acting as a stressor (Antelman et al. 1980; Assis et al. 2009; Barr et al. 2010; Wemm and Sinha 2019). Augmented release of corticosterone, suggesting increased HPA axis responsiveness, in parallel with immunosuppressive effects, more pronounced in the AMPH after withdrawal group, also suggests that this drug acts as a stressor which facilitates immunosuppression and sensitizes the release of corticosterone, immunosuppressive hormone. It is possible that the chronic, random AMPH treatment, as well as the long-term withdrawal phase (discontinuation of AMPH intake), act as chronic stressors causing much stronger immunosuppressive effects in response to the subsequent AMPH injection (subsequent stressor) in the AMPH after withdrawal group rather than after the chronic AMPH treatment alone.

The AMPH-induced decreases in the number of peripheral and spleen white blood cells, that we observed in the present study, may result from changes in the distribution of leukocytes, including lymphocytes. The HPA axis activation by AMPH and an increased secretion of glucocorticoids are major pathways through which the central nervous system modulates functions of the immune system (Dhabhar et al. 1995; Connor 2004; Wrona 2006; Gomez-Roman et al. 2016). Therefore, activation of the HPA axis may be involved in regulation of the immune cell distribution and function following AMPH exposure. Ligeiro-Oliveira et al. (2004) revealed that acute AMPH treatment (1 mg/kg) changed HPA-axis responsiveness to the stress condition imposed by the immunization decreasing lung and blood leukocyte numbers via corticosterone actions on bone marrow activity. The results of AMPH-induced increased plasma corticosterone concentration in the current study, with the highest level of this hormone and the most decreased immune response following challenge to the drug after withdrawal from chronic AMPH treatment, also support this opinion.

It should be pointed out that both an increased number of natural killer (NK) cells present in the blood and increased ability of blood Th2 subpopulation of TCD4^+^ lymphocytes to produce IL-4 along with decreases in the number of circulating and spleen leukocytes, total lymphocytes, T, B lymphocyte populations and production of IFN-ɤ by Th1 subpopulation of TCD4^+^ lymphocytes after the chronic, random AMPH challenge alone, were observed. This dual mechanism of AMPH influence on the blood immune response, in particular, an enhancement of NK cell number, could be related to the activation of the central catecholamines (Rothman et al. 2001) that are known to stimulate NK cell activity (NKCC) and NK cell number (Dhabhar et al. 2012), and are involved in the process of leukocyte recirculating through immune organs or other regions. Recirculation is the main and most probable cause of NKCC and leukocyte enhancement or decrease in the peripheral blood (Lang et al. 2003). However, opinions concerning the influence of AMPH on NKCC and NK cell number are not uniform. House et al. (1994, 1995) and Nunez-Iglesias et al. (1996) described suppressive effects of AMPH on NKCC. In addition, according to Saito et al. (2008), single or repeated METH injections reduced NKCC of spleen lymphocytes, especially after 5 injections, whereas with 10 METH injections the NKCC and leukocytes recovered to the level of controls. In contrast, Swerdlow et al. (1991) pointed out the possibility of the enhancement of NKCC and an increase in NK cell number in chronic AMPH users. Recently, Camacho et al. (2019) found fourfold-increase in the NK cell-specific transcript in the circulating blood when repeated injections of AMPH produced hyperthermia, indicating increases in NK cells. Furthermore, we previously reported (Wrona et al. 2005; Glac et al. 2006) that acute exposure to AMPH induced an increase in the blood and a decrease in the spleen NKCC and NK cell number, concomitantly with an increased plasma corticosterone level in rats. It suggests that AMPH-induced increase in blood NK cell numbers and function might have occurred due to the entry of NK cells into the circulation from the spleen. Similar effects have been noticed in the present experiment, with the elevation of the blood NK cell number after the chronic, random AMPH treatment alone. This transient elevation in the blood NK cell number could be related to the mobilization of these cells from the spleen into the peripheral blood as a compensatory mechanism, preventing reduction of these effector cells in the peripheral blood under AMPH-induced sympathetic system activation and/or high plasma CORT concentration. In fact, we observed the increased plasma corticosterone level in the AMPH-treated groups in this study.

In the present study, the long-term withdrawal from the AMPH treatment phase was associated with normalization of the blood immune response, except for significantly decreased blood TCD4^+^ and TCD8^+^ lymphocyte numbers. This recovery of the immune parameters may be considered to be a rebound from AMPH-induced suppression of the immune response. On the other hand, AMPH-induced immunostimulation (increased ability of blood lymphocyte to produce IL-4 and elevate NK cell number) observed after chronic AMPH treatment alone, may indicate that these immunostimulative effects are transient in nature. We have not observed such immunostimulative effects after the single injection of AMPH following withdrawal from the drug.

With respect to AMPH-induced changes in the blood lymphocyte function, the IFN-γ/IL-4 cytokine production profile, manifested by suppressed IFN-ɤ and increased IL-4 production in the blood after the chronic, random drug treatment, also indicated that AMPH abuse resulted in severe dysregulation in the peripheral immune response, leading to imbalanced expression in cytokines amplifying anti-inflammatory responses/activation by stimulating release of anti-inflammatory cytokines. These observations are in agreement with the results of Boyle and Connor (2007) who reported MDMA-induced suppression of the innate pro-inflammatory response evoked by cytokine IFN-γ. These and our results support data on long-term AMPH-induced suppression of the peripheral blood inflammatory response. According to Elenkov and Chrousos (1999), change in pro/anti-inflammatory cytokines and Th1/Th2 subsets of TCD4 lymphocytes, increased the risk of disease development, in particular infectious diseases. In the present study, the suppressed ability of TCD4^+^ lymphocytes to produce of IFN-ɤ with the increased release of IL-4 along with the corresponding decreased number of TCD4^+^ cells after the chronic AMPH treatment alone, may be due to a marked decrease in Th1 lymphocyte subset. On the other hand, an increase in Th2 subset of the TCD4^+^ lymphocyte number and/or an elevated activity of Th2 cells to produce anti-inflammatory cytokine in the blood could be responsible for these effects. It suggests that although the AMPH induced leucopenia, lymphopenia, and the decreased number of lymphocyte populations and T lymphocyte subsets, either it did not affect, or it facilitated the secretory activity of Th2 subpopulation of TCD4^+^ lymphocytes. Furthermore, in the present study, the AMPH treatment did not alter proportions between T lymphocyte subsets as indicated by the TCD4^+^/TCD8^+^ lymphocyte ratio. However, the greatest decrease in TCD4^+^ rather than TCD8^+^ cell number both after the chronic, random AMPH treatment alone and in the AMPH withdrawal groups, was noticed.

## Summary

In conclusion, the present data shows how amphetamine (AMPH) impacts the peripheral inflammatory response long-term. The results obtained indicate for the first time that the random AMPH exposure alone and acute (single injection) AMPH challenge following the long-term withdrawal syndrome induced long-term immunosuppressive effects, similar to those occurred during the stress response, and sensitized the peripheral immunosuppressive response of the rat to the disinhibitory effects of this stressor. Some aspects of the cell-mediated immunity were diminished as indicated by the decreased number of peripheral leukocytes, T, B lymphocytes, TCD4^+^ and TCD8^+^ lymphocyte subsets, granulocytes, monocytes, dysregulated cytokine profile, with the activation of anti-inflammatory and corticosterone response. These effects could implicate the risk of the development of pronounced immunodeficiencies and thus an increase in incidence of infectious diseases in case of a long-term AMPH abuse. The pronounced long-term changes produced by AMPH provide further insight into the link between this psychoactive drug’s use and its consequences to health outcomes in the long term (after AMPH withdrawal and the subsequent drug exposure) in regards to the increased risk of a disease and what correcting immune changes will be useful for the therapy of AMPH addiction.
